# Electroacupuncture for postoperative ileus after laparoscopic surgery on colorectal cancer: study protocol for a randomized controlled trial

**DOI:** 10.1186/s13063-021-05564-3

**Published:** 2021-09-09

**Authors:** Jia-Kai Shao, Qian Liu, Wei Pei, Yu Wang, Na-Na Yang, Ling-Yu Qi, Jin Huang, Jing-Wen Yang, Cun-zhi Liu

**Affiliations:** 1 International Acupuncture and Moxibustion Innovation Institute, School of Acupuncture-Moxibustion and Tuina, Beijing University of Chinese Medicine, No. 11, Bei San Huan Dong Lu, Chaoyang District, Beijing, 100029 China; 2 Department of Colorectal Surgery, Chinese Academy of Medical Sciences Cancer Institute and Hospital, Beijing, China

**Keywords:** Postoperative ileus, Electroacupuncture, Single acupoint, Randomized controlled trial

## Abstract

**Background:**

Postoperative ileus (POI) occurs in almost all patients after abdominal laparoscopic surgery, resulting in complications and increasing the length of hospitalization. Electroacupuncture has been used as an alternative therapy for gastrointestinal dysfunction, but its efficacy for POI is inconclusive. The study is designed to determine whether electroacupuncture can accelerate recovery from POI.

**Methods/design:**

This study is a three-arm, randomized controlled trial. A total of 105 patients will be randomized into a group receiving electroacupuncture at Tianshu (ST25), a group receiving electroacupuncture at Zusanli (ST36), or a control group in a 1:1:1 ratio. Patients in the electroacupuncture groups will receive electroacupuncture treatment for 4 days from the first day after surgery. The primary outcome consists of the time to first flatus and the time to first defecation. Secondary outcomes include the time to first tolerance of liquid and semiliquid food; the length of the hospital stay; postoperative pain, nausea, and vomiting; abdominal distension; the time to first get out of bed; and postoperative complications. The outcomes will be assessed by the patients themselves every day during hospitalization. Surgeons, nurses, assessors, and statisticians will be blinded to the group assignments. Patients in the two electroacupuncture groups, but not in the control group, will be blinded to the group assignments. The acupuncturists will not be blinded.

**Discussion:**

The aim of this trial is to provide a nonpharmacological therapy for POI and may provide evidence of the effect of electroacupuncture at ST25 or ST36 on POI.

**Trial registration:**

Chinese Clinical Trial Registry ChiCTR1900027466. Registered on 14 November 2019.

**Supplementary Information:**

The online version contains supplementary material available at 10.1186/s13063-021-05564-3.

## Background

Postoperative ileus (POI) is defined as a transient inhibition of gastrointestinal motility caused by abdominal surgery, especially colorectal cancer surgery [1, 2]. POI occurs in all postoperative patients after abdominal surgical procedures, even if minimally invasive techniques are used, and lasts for 2 to 4 days [3]. POI has a significant impact on postoperative recovery, with symptoms such as abdominal distension, pain, nausea, vomiting, delayed exhaust and defecation, the disappearance of bowel sounds, and intolerance to oral feeding [4]. POI is a self-limited functional gastrointestinal disease but decreases the quality of postoperative life and causes serious complications [5]. In addition, POI can lead to longer hospital stays and higher healthcare costs [6–8].

The Food and Drug Administration (FDA) approved alvimopan to treat POI in 2008. However, the efficacy and economic benefits of this drug remain controversial [9–12]. Some clinical trials have shown the effectiveness of several interventions, including the placement of a nasogastric tube (NGT) [13], minimally invasive surgery [14], and the use of alvimopan [15], coffee [16], and chewing gum [17], for treating POI. However, the consensus is that the evidence supporting these treatments is not strong [18, 19]. Therefore, it is necessary to find other effective therapies.

Electroacupuncture is a nonpharmaceutical intervention that is commonly used to treat various functional gastrointestinal disorders [20, 21]. Systematic reviews have found that electroacupuncture can improve POI after colorectal cancer surgery [22, 23]. Multiple acupoints were used in the randomized controlled trials included in the reviews [24–26]. Using acupoint combinations may increase the infection rate of postoperative patients, who are at a high risk of infection [27–29]. Moreover, the effect of acupoint combinations may be weaker than that of a single acupoint for gastrointestinal dysfunction [30]. Thus, using a single acupoint may be a better choice for POI than acupoint combinations. ST25 (Tianshu) and ST36 (Zusanli) are located on the stomach meridian and are two commonly used acupoints in treating POI [31]. However, the effects of electroacupuncture at ST25 or ST36 on treating POI are unclear.

We assume that electroacupuncture at ST25 or ST36 can promote recovery from POI. Accordingly, we have designed a pilot trial to evaluate the therapeutic potential of electroacupuncture at ST25 (EA A) or ST36 (EA B) compared to a control on POI after laparoscopic surgery for colorectal cancer.

The hypotheses are as follows.

H0: Effect of EA A or EA B = effect of the control group

H1: Effect of EA A or EA B ≠ effect of the control group

## Methods/design

### Study design

**Fig 1:**
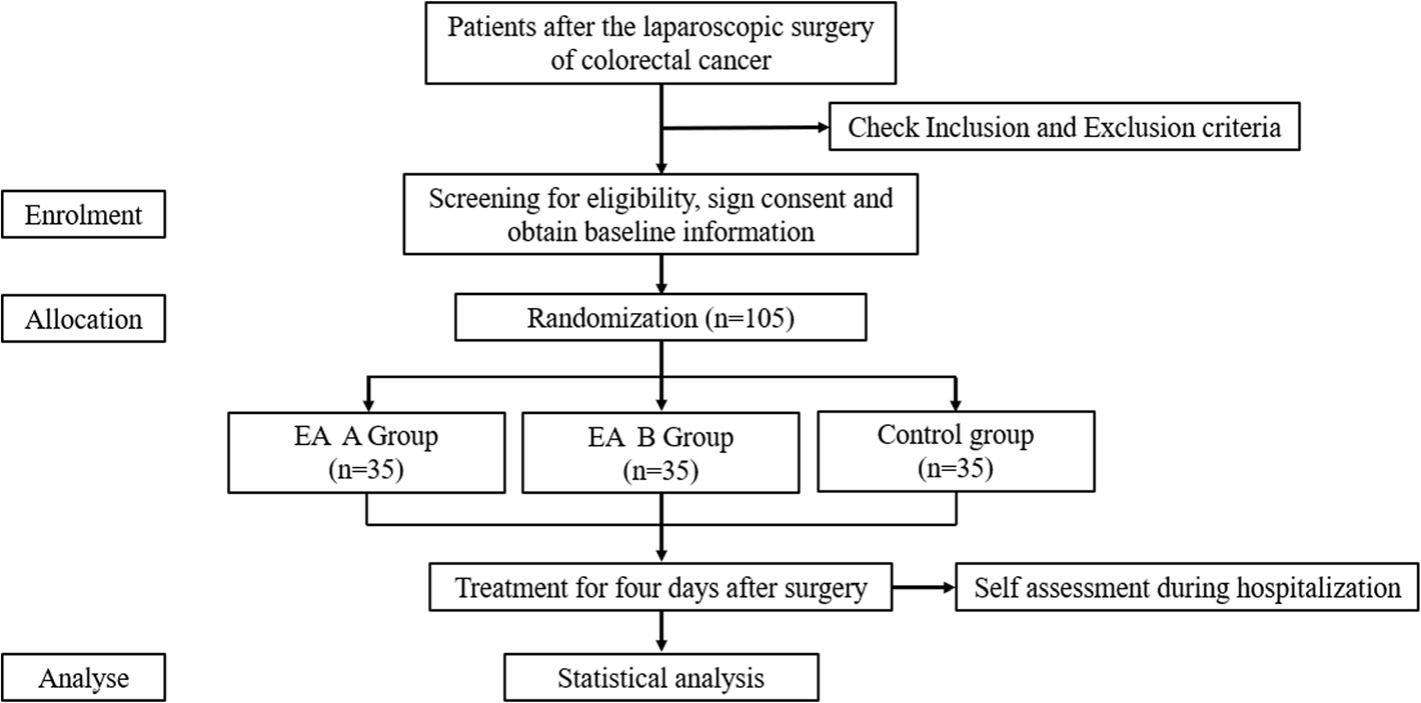
EA A Group: electroacupuncture at ST25 (Tianshu acupoint); EA B Group: electroacupuncture at ST36 (Zusanli acupoint)

This single-center, three-arm, prospective randomized trial will be conducted at the Cancer Hospital of the Chinese Academy of Medical Science. The protocol has been registered on chictr.org.cn (No. ChiCTR1900027466) and reported in full accordance with the SPIRIT guidelines. Figure 1 is a flow diagram of the trial.

### Population, recruitment, and ethics

Patients undergoing laparoscopic colorectal surgery will be informed of the intervention measures, intervention time and other trial procedures through advertisements in the hospital. Patients who meet the criteria for inclusion and exclusion after surgery will be provided with the aforementioned information again. Then, each patient will be asked to sign an informed consent form and recruited if he/she agrees to participate in the trial before randomization. The study investigators will be responsible for recruiting patients and obtaining informed consent. The study protocol has been approved by the Ethics Committee of Beijing University of Chinese Medicine and conforms to the Declaration of Helsinki (No. 2019BZHYLL0207).

#### Inclusion criteria

1. Age above 18 years;
2. Laparoscopic colorectal surgery has been performed;
3. Abdominal surgery has been performed for the first time;
4. American Society of Anesthesiologists (ASA) grade I–III;
5. Willing to provide written informed consent.

#### Exclusion criteria

1. Laparoscopy needs to be synchronized with other surgeries;
2. Laparoscopy will be followed by open surgery;
3. Intraoperative and postoperative complications requiring intensive care;
4. Taking a long-term medication that affects bowel function, such as Chinese herbs, vitamins or iron sulfates, within a month before surgery;
5. Has received acupuncture within a month before surgery;
6. Has a pacemaker;
7. History of syncope or epilepsy;
8. Epidural anesthesia during surgery;
9. Participation in other clinical studies within the last 3 months;
10. Has stoma creation.

### Randomization and allocation concealment

Patients will be randomly assigned to the EA A, EA B, and control groups in a 1:1:1 ratio. The randomization sequence will be generated by an independent statistician who will not participate in the study using block randomization in SAS 9.3 software. The treatment allocation code will be placed in an opaque envelope labeled with a sequential number. After informed consent is obtained from an eligible patient, the acupuncturist will receive an opaque envelope and begin electroacupuncture treatment on the two EA groups.

### Masking

Patients in the two EA groups, hospital staff (surgeons and nurses) assessors, and statisticians will be blinded to the group assignments, but the patients in the control group will not be blinded because of the particularity of the treatment. The acupuncturists will not be blinded.

### Intervention

#### Electroacupuncture groups (EA A and EA B)

The acupoints in the two EA groups are Tianshu (ST25) and Zusanli (ST36) (which will be located according to the WHO Standard presented in Table 1) [32]. Patients in the EA groups will receive acupuncture treatment at bilateral single acupoints using disposable stainless steel needles, 0.30 mm × 40 mm (Hwato, Suzhou, China). Only acupuncturists with more than 5 years of experience will be allowed to perform the intervention. Acupuncture stimulation will be performed for at least 10 s to achieve “deqi” (a radiating needle sensation accompanied by soreness and numbness), after which a pair of electrodes from an electric stimulator (HANS-200A acupoint nerve stimulator, Nanjing Jisheng Medical Co, Ltd) will be connected to the needle handles at ST25 or ST36. An electroacupuncture waveform with a 2/100-Hz density alternating wave will be adopted, and the intensity of the electric current will be increased up to a level that the patient can perceive and tolerate. The EA group will be treated for 30 min once a day for a total of 4 days from the first day after surgery. Acupuncture interventions will be recorded by the study investigators after each session to ensure patient compliance. The intervention plan is set for 4 days because the pathological status of POI lasts for 2–4 days [33]. In addition, all patients will receive routine standard postoperative care (RSPC).

**Table 1:** Location of ST25 and ST36

Acupoint	Location
**Tianshu (ST25)**	On the upper abdomen, 2 cun lateral to the center of the umbilicus.
**Zusanli (ST36)**	On the anterior aspect of the leg, on the line connecting ST35 and ST41, 3 cun inferior to ST35, locate on the tibialis anterior muscle.

#### Routine standard postoperative care (RSPC)

1. Multimodal pain management (use of nonopioid analgesics).
2. Patient-controlled anesthesia (PCA).
3. Sips of water after the patient has first flatus.
4. Intake of protein and energy-rich nutritional supplements.
5. Mobilization within 24–48 h: the patient will be instructed to ambulate at least once for any length of time between 24 and 48 h after surgery.

#### Control group

Patients in the control group will receive RSPC without acupuncture. At the end of the trial, if a patient is willing to contact us, we will give free acupuncture treatment.

### Primary outcome

The primary outcome is a composite endpoint consisting of the time to first flatus and the time to first defecation [34], which are defined as the time from the end of the operation to the first exhaust and defecation, respectively, and will be recorded in hours in the case report form by patients under the supervision of an assessor during hospitalization. The minimum clinical significance of the primary outcome is 12 h [27].

### Secondary outcomes

Secondary outcomes will include the length of the hospital stay; the time to first tolerance of liquid and semiliquid food; postoperative pain; postoperative use of analgesics; postoperative nausea and vomiting; the time to first walk independently; postoperative distension of the abdomen; and adverse events.

The length of the hospital stay is defined as the time from the end of the operation to discharge. The time to first tolerance of liquid and semiliquid food is defined as the time from the end of the operation to the first tolerance of the aforementioned two kinds of food without gastrointestinal adverse reactions. The time to first walk independently is defined as the time from the end of the operation to the first time the patient can get out of bed and ambulate. Postoperative pain will be evaluated regularly at three fixed times per day using the visual analog scale (VAS, from 0 to 10), and the three scores will be averaged. The higher the score is, the more severe the indication of pain and nausea is. Vomiting, nausea, and abdomen distension will be counted once, daily. The abovementioned outcomes will be recorded by the patient on a daily assessment form (DSF) during hospitalization.

Adverse events include postoperative complications and adverse acupuncture events. Postoperative complications, such as fever, intestinal edema, and intestinal anastomotic fistula, will be recorded and graded according to the Clavien-Dindo classification (Table 2) by the assessors. If patients have serious complications after surgery, the participants will be asked to discontinue the trial, and the doctor will administer appropriate treatment. Adverse acupuncture events include subcutaneous hematoma, continuous acupuncture afterfeeling, and itching at the sites of needle insertion and will be recorded by the assessors.

**Table 2:** Clavien-Dindo classification

**Grade 1**	Any deviation from the normal postoperative course without the need for pharmacologic treatment or surgical, endoscopic, and radiologic interventions. Allowed therapeutic regimens are drugs as antiemetics, antipyretics, analgetics, and diuretics, and electrolytes and physiotherapy. This grade also includes wound infections opened at the bedside
**Grade 2**	Requiring pharmacologic treatment with drugs other than such allowed for grade I complications. Blood transfusions and total parenteral nutrition are also included
**Grade 3**	Requiring surgical, endoscopic, or radiologic intervention
**3a**	Intervention not under general anesthesia
**3b**	Intervention under general anesthesia
**Grade 4**	Life-threatening complication (including CNS complications) requiring IC/ICU management^a^
**4a**	Single organ dysfunction (including dialysis)
**4b**	Multiple organ dysfunction
**Grade 5**	Death as a result of complications

*Abbreviations*: *CNS* central nervous system, *IC* intermediate care, *ICU* intensive care unit

^a^Brain hemorrhage, ischemic stroke, or subarachnoidal bleeding but excluding transient ischemic attacks

Handling of biological specimens is not applicable to this trial as none will be collected for any outcomes.

**Fig 2:**
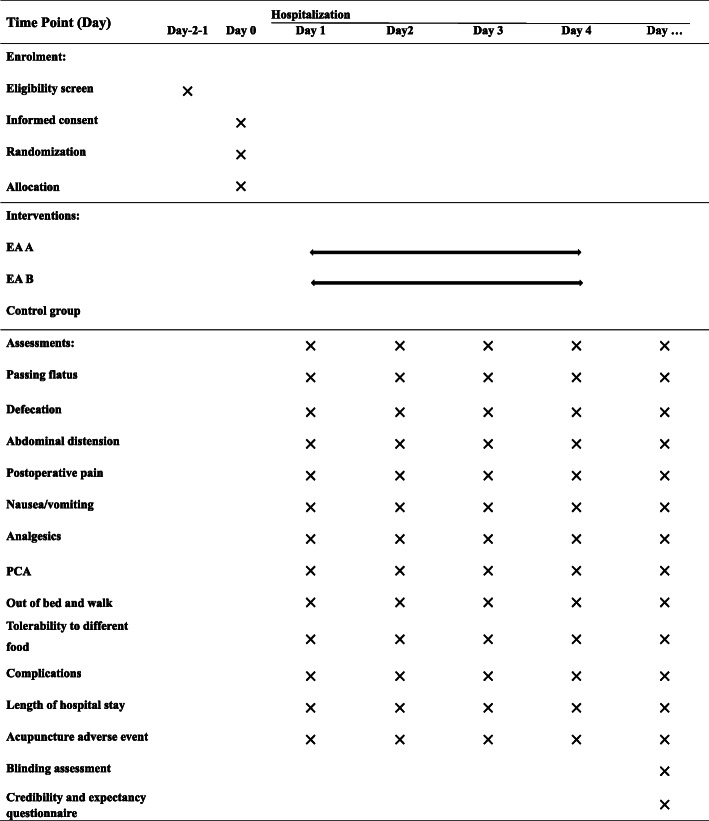
Schedule of enrollment, intervention, and assessment

The DSF is shown in Table 3. Figure 2 shows the schedule of enrollment, intervention, and assessment.

**Table 3:**
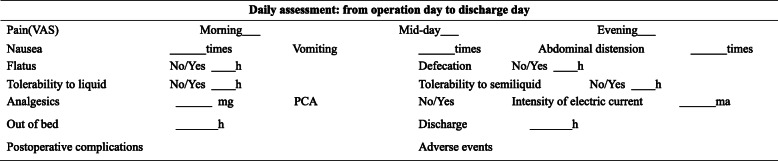
Daily assessment form during postoperative hospitalization

*VAS* visual analog scale, *PCA* patient controlled anesthesia

### Data management

All data will be collected in case report forms (CRFs) that have been created by study members and will be entered into electronic record form by two independent researchers to ensure the accuracy of the data from the operation day to the discharge day. The investigators will continue to collect data on patients in the ward who have fallen off treatment for as long as possible until the end of the trial (discharge). The data collectors will be trained to fill out the CRFs to ensure the accuracy of the data. Paper files, including the original CRFs (including the baseline data and DSF data) and consent forms, will be kept for more than 5 years after publication. All final data will only be accessed by the study staff and kept strictly confidential. Readers and reviewers who have questions or want to check the original data can contact the corresponding author. Private patient information, including names, home addresses, and telephone numbers, will be kept confidential at all times.

Moreover, an independent Data and Safety Monitoring Board (DSMB) will be set up to review the trial data every month and determine whether the patient needs to discontinue from this trial in the event of complications and seek medical attention. The members of the DSMB are selected and appointed by the sponsor and include physicians and ethicists who are not involved in the study. The role of the DSMB is to help sponsors maintain a rigorous study design, give appropriate attention to subject protection, review and evaluate clinical efficacy and safety data collected during the study, and evaluate cumulative reports of serious adverse events.

### Sample size

The primary outcome consists of the time to first flatus and the time to first defecation. Based on previous trials [24, 25] and clinical experience, the time to first flatus will be assumed to be 48 h and 64.8 h for the EA group and the control group, respectively, with a standard deviation of 21.6. Given a 2.5% two-sided significance, 80% power, and 10% drop rate, a sample size of 105 patients will be recruited, with 35 patients in each group. The time to first defecation will be assumed to be 86 h and 122 h for the EA group and the control group, respectively, with a standard deviation of 45. Given a 2.5% two-sided significance, 80% power, and 10% drop rate, a sample size of 90 patients will be recruited, with 30 patients in each group. In conclusion, 105 patients, with 35 patients in each group, will be recruited.

### Statistical analysis

All statistical analyses will be performed according to the intention-to-treat (ITT) principle, which includes patients who will have received electroacupuncture at least once or who will have completed DSF for at least 1 day.

All paper data will be input into the electronic CRF and analyzed by SPSS 20. Measurement data will be presented as the mean ± standard deviation (M ± SD) or median and quartile spacing, and counting data will be presented as the composition ratio and percentage. Demographic and other baseline data will be compared by ANOVA and the chi-square test. The primary outcome and the time endpoints of the secondary outcomes will be compared by the Kaplan-Meier Breslow (generalized Wilcoxon) test. The incidence of pain, nausea, and abdominal distension; the frequency of vomiting; and the incidence of complications will be analyzed by the chi-square test or Mann-Whitney *U* test. Factors that may affect the results such as the stage and metastasis of colorectal will be recorded in the baseline. If these factors are unbalanced at baseline, we will use these factors as stratification factors to control the potential influence. The statistically significant *P* test level will be less than 0.025 for the primary outcome and less than 0.05 for the secondary outcomes. The primary outcome will be considered to be statistically different when both the first flatus time and the first defecation time are statistically different between the groups. The missing data will be filled using the last observation carry-over method. No additional, subgroup or interim analyses are planned for the trial.

## Discussion

POI is a pathological state that always occurs after colorectal surgery and inflicts a considerable financial burden on patients and society. This trial will evaluate the effects of performing EA at ST25 or ST36 in accelerating the POI recovery time.

Although several trials have been conducted, the consensus [19] is that acupuncture is not a recommended treatment for POI, which may be due to the low reliability of trials. This trial fully meets methodological requirements and achieves full randomization of patients and blinding for patients, hospital staffs, outcome assessors and statisticians.

Actual clinical results show that postoperative patients are more likely to accept acupuncture with fewer acupoints, which can reduce the discomfort caused by acupuncture. Thus, we have chosen ST25 or ST36 as treatment acupoints, which have already been proven to be effective for gastrointestinal dysfunction [35–37]. Our aim is to observe whether EA at a single acupoint can promote recovery from POI.

The pathological state of POI lasts for a maximum of 4 days after surgery [33]. Our intervention will last for 4 days after surgery to ensure that every patient is treated with electroacupuncture during the pathological state. In addition, EA with 2/100-Hz stimulation has been proven to be effective in reducing postoperative incision and visceral pain [38, 39].

A limitation of the trial is that visible acupoints in patients in the two groups receiving acupuncture may break blindness. Another limitation is that as all patients will receive RSPC which is based on a protocol for enhancing recovery after surgery [40], it may lead to a ceiling effect, and the efficacy of electroacupuncture may be negatively affected. However, we hope the trial will provide more reliable evidence than previous studies and clarify the value of electroacupuncture as a treatment for POI.

## Supplementary Information

**Additional file 1.**

## Data Availability

The generated and analyzed datasets for the study will be publicly available from the corresponding author on reasonable request. The results will be published in peer-reviewed international journals.

## References

[1] Harnsberger Cristina R, Maykel Justin A, Karim A (2019). Postoperative ileus. Clin Colon Rectal Surg.

[2] Avo A, Nunoo-Mensah Joseph W, Swarna B (2008). Prolonged postoperative ileus-definition, risk factors, and predictors after surgery. World J Surg.

[3] Boeckxstaens GE, de Jonge WJ (2009). Neuroimmune mechanisms in postoperative ileus. Gut..

[4] Stakenborg N, Gomez-Pinilla PJ, Boeckxstaens GE (2017). Postoperative ileus: pathophysiology, current therapeutic approaches. Handb Exp Pharmacol.

[5] Venara A, Neunlist M, Slim K, Barbieux J, Colas PA, Hamy A, Meurette G (2016). Postoperative ileus: pathophysiology, incidence, and prevention. J Visc Surg.

[6] Kraft MD (2008). Methylnaltrexone, a new peripherally acting mu-opioid receptor antagonist being evaluated for the treatment of postoperative ileus. Expert Opin Investig Drugs.

[7] Kauf Teresa L, Svatek Robert S, Gilad A (2014). Alvimopan, a peripherally acting μ-opioid receptor antagonist, is associated with reduced costs after radical cystectomy: economic analysis of a phase 4 randomized, controlled trial. J Urol.

[8] Henrik K (2008). Postoperative ileus--an update on preventive techniques. Nat Clin Pract Gastroenterol Hepatol.

[9] Xu LL, Zhou XQ, Yi PS, Zhang M, Li J, Xu MQ (2016). Alvimopan combined with enhanced recovery strategy for managing postoperative ileus after open abdominal surgery: a systematic review and meta-analysis. J Surg Res.

[10] Nair A (2016). Alvimopan for post-operative ileus: what we should know?. Acta Anaesthesiol Taiwanica.

[11] Adam MA, Lee LM, Kim J, Shenoi M, Mallipeddi M, Aziz H, Stinnett S, Sun Z, Mantyh CR, Thacker JKM (2016). Alvimopan provides additional improvement in outcomes and cost savings in enhanced recovery colorectal surgery. Ann Surg.

[12] Keller DS, Flores-Gonzalez JR, Ibarra S, Mahmood A, Haas EM (2016). Is there value in alvimopan in minimally invasive colorectal surgery?. Am J Surg.

[13] Fabio P, Fausto R, Daniele M (2014). Naso-gastric or naso-jejunal decompression after partial distal gastrectomy for gastric cancer. Final results of a multicenter prospective randomized trial. Gastric Cancer.

[14] van Bree SHW, van Bree S, Vlug Malaika S (2011). Faster recovery of gastrointestinal transit after laparoscopy and fast-track care in patients undergoing colonic surgery. Gastroenterology..

[15] Lee Cheryl T, Chang Sam S, Kamat Ashish M (2014). Alvimopan accelerates gastrointestinal recovery after radical cystectomy: a multicenter randomized placebo-controlled trial. Eur Urol.

[16] Cornwall Hannah L, Edwards Ben A, Curran John F (2020). Coffee to go? The effect of coffee on resolution of ileus following abdominal surgery: A systematic review and meta-analysis of randomised controlled trials. Clin Nutr.

[17] Edna PGM, Rachel R, Porfírio Gustavo J (2016). Chewing gum for enhancing early recovery of bowel function after caesarean section. Cochrane Database Syst Rev.

[18] Hedrick Traci L, McEvoy Matthew D, Mythen Michael Monty G (2018). American Society for Enhanced Recovery and Perioperative Quality Initiative Joint Consensus Statement on postoperative gastrointestinal dysfunction within an enhanced recovery pathway for elective colorectal surgery. Anesth Analg.

[19] Daniel G, Olivier G, Martin H (2017). Postoperative ileus: in search of an international consensus on definition, diagnosis, and treatment. Langenbeck's Arch Surg.

[20] World Health Organization. Programme on Traditional Medicine. WHO traditional medicine strategy 2002-2005. World Health Organization; 2002. https://apps.who.int/iris/handle/10665/67163.

[21] Chen JDZ, Ni M, Yin J (2018). Electroacupuncture treatments for gut motility disorders. Neurogastroenterol Motil.

[22] Yihong L, May Brian H, Lin ZA (2018). Acupuncture and related therapies for treatment of postoperative ileus in colorectal cancer: a systematic review and meta-analysis of randomized controlled trials. Evid Based Complement Alternat Med.

[23] Yi-Hua L, Dong G-T, Yang Y (2017). Effectiveness of acupuncture for early recovery of bowel function in cancer: a systematic review and meta-analysis. Evid Based Complement Alternat Med.

[24] Ng Simon SM, Leung Wing W, Mak Tony WC (2013). Electroacupuncture reduces duration of postoperative ileus after laparoscopic surgery for colorectal cancer. Gastroenterology..

[25] Deng G, Douglas WW, Jose G (2013). A phase II, randomized, controlled trial of acupuncture for reduction of postcolectomy ileus. Ann Surg Oncol.

[26] Yang Y, Hong-Qun Z, Zhao L (2017). Comparison of efficacy of simo decoction and acupuncture or chewing gum alone on postoperative ileus in colorectal cancer resection: a randomized trial. Sci Rep.

[27] Rollins Katie E, Hannah J-E, Acheson Austin G (2019). The role of oral antibiotic preparation in elective colorectal surgery: a meta-analysis. Ann Surg.

[28] Scarborough John E, Jessica S, Craig KK (2017). Associations of specific postoperative complications with outcomes after elective colon resection: a procedure-targeted approach toward surgical quality improvement. JAMA Surg.

[29] Sung-Min P, Won-Jeong K, Je-Ho M (2016). Adverse events associated with acupuncture: a clinicopathologic review. Int J Dermatol.

[30] Qingguang Q, Wang H, Kun L (2013). Effects of acupuncture on intestinal motility: agonism and antagonism. World Chin Med.

[31] Ya-Quan H, Jian-feng T, Mei-lin J (2019). Analysis of acupoint selection rules in the treatment of postoperative ileus by acupuncture. Jilin J Chin Med.

[32] WHO Regional Office for the Western Pacific. WHO Standard Acupuncture Point Locations in the Western Pacific Region. Manila: World Health Organization; 2008. ISBN 9789290613831.

[33] Delaney CP (2004). Clinical perspective on postoperative ileus and the effect of opiates. Neurogastroenterol Motil.

[34] Van Bree SHW, Bemelman Willem A, Hollmann Markus W (2014). Identification of clinical outcome measures for recovery of gastrointestinal motility in postoperative ileus. Ann Surg.

[35] Zhishun L, Shiyan Y, Wu J (2016). Acupuncture for chronic severe functional constipation: a randomized trial. Ann Intern Med.

[36] Liang C, Wang K-Y, Gong M-R, Li Q, Yu Z, Xu B (2018). Electro-acupuncture at ST37 and ST25 induce different effects on colonic motility via the enteric nervous system by affecting excitatory and inhibitory neurons. Neurogastroenterol Motil.

[37] Jun-Fan F, Jian-Qiao F, Xiao-Mei S (2017). Electroacupuncture treatment partly promotes the recovery time of postoperative ileus by activating the vagus nerve but not regulating local inflammation. Sci Rep.

[38] Teixeira FM, Castro LL, Ferreira RT, Pires PA, Vanderlinde FA, Medeiros MA (2012). High-frequency electroacupuncture versus carprofen in an incisional pain model in rats. Braz J Med Biol Res.

[39] Jingzhu Z, Shiying L, Wang Y (2017). Inhibitory effects and mechanisms of electroacupuncture via chronically implanted electrodes on stress-induced gastric hypersensitivity in rats with neonatal treatment of iodoacetamide. Neuromodulation..

[40] Olle L, Michael S, Fearon Kenneth C (2017). Enhanced recovery after surgery: a review. JAMA Surg.

