# Supplementary material for: Electroacupuncture for postoperative ileus after laparoscopic surgery on colorectal cancer: study protocol for a randomized controlled trial
Source: Trials. 2021 Sep 9;22:610. doi: 10.1186/s13063-021-05564-3 (PMC8428035; doi:10.1186/s13063-021-05564-3)
Supplement: Supplementary file 1 — Additional file 1. [file 13063_2021_5564_MOESM1_ESM.docx]

Supplemental table

| Registration number | ChiCTR1900027466 | | |
| --- | --- | --- | --- |
| Registration Status | 1008001 Prospective registration | | |
| Public title | Electroacupuncture for postoperative ileus after colorectal cancer surgery: a randomized controlled clinical study. | | |
| Scientific title | Electroacupuncture for postoperative ileus after colorectal cancer surgery: a randomized controlled clinical study. | | |
| Applicant | Jiakai Shao | Study leader | Cunzhi Liu |
| Applicant telephone | +86 18610915291 | Study leader's telephone | +86 15901261692 |
| Applicant E-mail | 785347789@qq.com | Study leader's E-mail | lcz_tg@126.com |
| Applicant address | 11 North Third Ring Road East, Chaoyang District, Beijing | Study leader's address | 11 North Third Ring Road East, Chaoyang District, Beijing |
| Applicant's institution | School of Acupuncture-Moxibustion and Tuina, Beijing University of Chinese Medicine. | | |

| Approved by ethic committee | | Yes | | |
| --- | --- | --- | --- | --- |
| Approved No. of ethic committee | | 2019BZHYLL0207 | Approved file of Ethical Committee | Ethical Approval Document |
| Name of the ethic committee | | Ethics Committee of Beijing University of Chinese Medicine | | |
| Date of approved by ethic committee | | 2019/07/01 | | |
| Contact Name of the ethic committee | | Lingling Qin | | |
| Contact Address of the ethic committee | | 11 North Third Ring Road East, Chaoyang District, Beijing | | |
| Primary sponsor's address | 11 North Third Ring Road East, Chaoyang District, Beijing | | | |
| Secondary sponsor | \| Country: \| China \| Province \| Beijing \| City: \|  \| \| --- \| --- \| --- \| --- \| --- \| --- \| \| Institution hospital \| Beijing University of Chinese Medicine \| Address \| 11 North Third Ring Road East, Chaoyang District \| \|  \| | | | |
| Source(s) of funding | The National Science Fund for Distinguished Young Scholars (81825024) | | | |
| Target disease | postoperative ileus | | | |
| Study type | Interventional study | | | |
| Objectives of Study | To evaluate the efficacy and safety of zusanli point and tianshu point for postoperative ileus. | | | |
| Study design | Parallel | | | |
| Inclusion criteria | 1. Aged above 18 years, male or female; 2. Laparoscopic colorectal cancer surgery was performed; 3. Abdominal surgery was performed for the first time; 4. American Society of Anesthesiologists(ASA) grading I–III. 5. Willing to sign written informed consent. | | | |
| Exclusion criteria | 1. Need to be synchronized with other surgeries; 2. Go from laparoscopy to open surgery; 3. Intraoperative and postoperative complications requiring intensive care; 4. Taking drugs such as Chinese herbs, vitamins or iron sulfates which affect bowel function within a month; 5. Acupuncture within a month; 6. Having pacemakers; 7. History of syncope or epilepsy; 8. Epidural anesthesia in surgery; 9. Participated in other clinical studies within the last 3 months. | | | |
| Study execute time | From2019/11/14 To 2020/11/30 | | | |

| Interventions | | \| Group \| Electroacupuncture at zusanli +routine standard postoperative care group \| \| Sample size: 35 \|  \| \| \| --- \| --- \| --- \| --- \| --- \| --- \| \| Group \| \| Electroacupuncture at tianshu +routine standard postoperative care group \| Sample size: 35 \| \|  \| \| Group \| \| No acupuncture group（Control group） \| Sample size: 35 \| \|  \| |
| --- | --- | --- | --- | --- | --- | --- | --- | --- | --- | --- | --- | --- | --- | --- | --- | --- | --- | --- | --- | --- |
| Countries of recruitment and research settings | \| Country \| China \| Province \| Beijing \| City: \|  \| \| --- \| --- \| --- \| --- \| --- \| --- \| \| Institution hospital \| Cancer Hospital Chinese Academy of Medical Sciences \| Level of the institution \| Tertiary A \| \| \| | |

| Outcomes | \| Outcome: time to first flatus after surgery \|  \| \| \| \| \|  \| \| --- \| --- \| --- \| --- \| --- \| --- \| --- \| \| Outcome: time to first defecation after surgery \|  \| \| \| \| \|  \| \| Outcome: length of hospital stay \|  \| \| \| \| \|  \| \| Outcome: time to tolerating oral diet \|  \| \| \| \| \|  \| \| Outcome: postoperative nausea per day(Visual Analogue Scale，VAS) \| \| \|  \| \| \|  \| \| Outcome: postoperative pain per day (Visual Analogue Scale, VAS) \| \|  \| \| \|  \| \| \| Outcome: time to first walk independently \|  \| \| \|  \| \| \| \| Outcome: postoperative complications（Clavien-Dindo） \| \| \| \| \| \| \| | | | | | |
| --- | --- | --- | --- | --- | --- | --- | --- | --- | --- | --- | --- | --- | --- | --- | --- | --- | --- | --- | --- | --- | --- | --- | --- | --- | --- | --- | --- | --- | --- | --- | --- | --- | --- | --- | --- | --- | --- | --- | --- | --- | --- | --- | --- | --- | --- | --- | --- | --- | --- | --- | --- | --- | --- | --- | --- | --- | --- | --- | --- | --- | --- | --- |
| Recruiting status | | | Recruiting | | | \| Participant age: above 18 years \| \| --- \| |
| Gender | | | | Both | |  |
| Randomization Procedure (please state who generates the random number sequence and by what method) | | | The blocked randomization sequence was prepared by a professional statistician with the SAS 9.3 software, who is not involved in assessment, treatment or analysis. | | | |
| Blinding | | Subjects in the two electroacupuncture group, data collectors statistical analysis were blinded .Subjects in the routine care group could not be blinded due to particularity. Acupuncturist were not blinded in this study. | | | | |
| Calculated Results ater the Study Completed public access | | Public | | | | |
| IPD sharing | | | | | Yes | |
| The way of sharing IPD”(include metadata and protocol, If use web-based public database, please provide the url) | | | | | Within 6 months after the trial complete, we will share IPD based on the ResMan platform (http://www.medresman.org.cn.) | |
| Data collection and Management (A standard data collection and management system include a CRF and an electronic data capture | | | | | The records of metadata are recorded through a CRF form, and then cross-checked and transcribed to an electronic database file based on Epidata software. All the data management is handled by a dedicated person. | |
